# Molecular Docking of Bacosides with Tryptophan Hydroxylase: A Model to Understand the Bacosides Mechanism

**DOI:** 10.1007/s13659-014-0031-5

**Published:** 2014-07-19

**Authors:** David Mary Rajathei, Jayakumar Preethi, Hemant K. Singh, Koilmani Emmanuvel Rajan

**Affiliations:** 1 Department of Bioinformtics, Bharathidasan University, Tiruchirappalli, 620024 India; 2 Department of Animal Science, School of Life Sciences, Bharathidasan University, Tiruchirappalli, 620024 India; 3 Laboratories of CNS Disorder, Learning & Memory, Division of Pharmacology, Central Drug Research Institute, Lucknow, 226031 India

**Keywords:** Serotonin, Tryptophan hydroxylase, Bacosides, Docking

## Abstract

Tryptophan hydroxylase (TPH) catalyses l-tryptophan into 5-hydroxy-l-tryptophan, which is the first and rate-limiting step of serotonin (5-HT) biosynthesis. Earlier, we found that TPH2 up-regulated in the hippocampus of postnatal rats after the oral treatment of *Bacopa monniera* leaf extract containing the active compound bacosides. However, the knowledge about the interactions between bacosides with TPH is limited. In this study, we take advantage of in silico approach to understand the interaction of bacoside-TPH complex using three different docking algorithms such as HexDock, PatchDock and AutoDock. All these three algorithms showed that bacoside A and A_3_ well fit into the cavity consists of active sites. Further, our analysis revealed that major active compounds bacoside A_3_ and A interact with different residues of TPH through hydrogen bond. Interestingly, Tyr235, Thr265 and Glu317 are the key residues among them, but none of them are either at tryptophan or BH4 binding region. However, its note worthy to mention that Tyr 235 is a catalytic sensitive residue, Thr265 is present in the flexible loop region and Glu317 is known to interacts with Fe. Interactions with these residues may critically regulate TPH function and thus serotonin synthesis. Our study suggested that the interaction of bacosides (A_3_/A) with TPH might up-regulate its activity to elevate the biosynthesis of 5-HT, thereby enhances learning and memory formation.

## Introduction

*Bacopa monniera* L. (Scrophulariaceae), commonly known as Brahmi, is a creeping plant with bitter taste found in marshy areas in India. It is used in the Indian system of ayurvedic medicine to enhance cognitive function. The leaf extract of *B. monniera* contains bioactive compounds bacosides A_1_–A_3_ [1–3], bacopasaponins A–G [4–7] and bacopasides I–V [8, 9]. A study claimed that bioactive compounds extracted from *B. monniera* improved acquisition, consolidation and retention of newly acquired behavior in rats [10]. Subsequent studies demonstrate that these compounds significantly attenuated the phenytoin [11], diazepam [12], hypobaric hypoxia [13], L-NNA [14], scopolamine [15], *m*CPBG [16] and d-galactose [17] induced memory impairments in rats. *B. monniera* treatment modulates various neurotransmitters such as acetylcholine (ACh), serotonin (5-hydroxytryptamine, 5-HT), gamma amino butyric acid (GABA), glutamate (Glu) and dopamine (DA) at different brain regions to enhance the cognitive functions [16, 18–24].

It has been documented that variations in serotonin metabolism and transmission is often associated with changes in short and long-term memory [25–28]. Biosynthesis of serotonin is regulated by the rate-limiting enzyme tryptophan hydroxylase (TPH) (EC. 1. 14. 16. 4), which converts l-tryptophan to 5-hydroxy-l-tryptophan using molecular oxygen, non-heme iron (II) with tetrahydrobiopterin (BH_4_) as a cofactor [29, 30]. There are two forms of TPH; TPH1 is responsible for most peripheral 5-HT, whereas, TPH2 is neuronal specific [31–34]. Isoforms of TPH (TPH1, TPH2) are highly homologous with an identity of 68–71 % across the phyla, and possess similar three-dimensional structure with respect to the location of amino acid residues where the substrate binds or the active site geometry [35]. TPH2 holds different kinetic properties like higher specificity for l-tryptophan but a lower catalytic efficiency. It has higher molecular weight than TPH1 because of the elongated N-terminal domain (approx. 56 kDa compared to TPH1 which is 51 kDa) and also possess an additional phosphorylation site [36]. Studies indicate that up-regulation of TPH expression leads to an enhancement of TPH activity and 5-HT synthesis [37, 38].

Recently, we found that the administration of *B. monniera* leaf extract in postnatal rats elevates the 5-HT level by up-regulating tryptophan hydroxylase-2 (TPH2) and serotonin transporter (SERT) expression [24]. Thus the elevated 5-HT level influences learning and memory by modulating other ACh and Glu, acting through the 5-HT_3_ receptor [16]. The structure of TPH with Fe and its cofactor BH_2_ complex have been documented [39]. However, the interaction between bacoside A and TPH was not studied yet. Based on the limitations, as a first attempt we adopted in silico approach to study the complex interactions. At this point, we used three different automatic molecular docking algorithms such as AutoDock, PatchDock and HexDock to identify interactions between the complexes. Through various interactions, molecular recognition occurs between protein and ligand, which is critical for identifying binding partners to the binding site. Our in silico study showed the interaction of bacosides (A_3_, A) with TPH, which is believed to be involved in the up-regulation of 5-HT synthesis.

## Results and Discussion

**Fig. 1:**
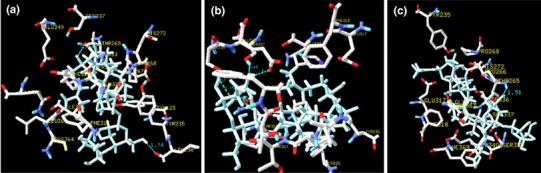
3D molecular structure from combination of TPH and bacoside A generated using HexDock (**a**), PatchDock (**b**) and AutoDock (**c**) showing the interactions with key residues

**Fig. 2:**
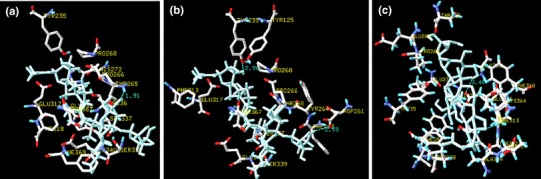
TPH and bacoside A complex 3D molecular structure generated using HexDoc (**a**), PatchDock (**b**) and AutoDock (**c**) showing the interactions with key residues Thr265, Tyr235 and Glu317 respectively

To uncover the structural basis of docking interactions between TPH and bacosides, we sought to determine the complex formation. Number of active compounds was identified for therapeutic targets based on the binding of ligand with protein, in which virtual screening and computational tools plays a major role [40, 41]. Bacosides are non-polar glycosides; lipid-mediated transportation by passive diffusion may facilitate transport across blood–brain barrier [8, 9, 42] and interact with TPH2. The general 3D molecular structure with combination of bacoside-TPH showed that the bioactive compounds present in the *B. monniera* leaf extract interact with TPH, which may up-regulate TPH2 expression [24]. In our study, we tested bacoside-TPH interactions in three different algorithms developed based on combinations of various scoring functions. This would help us to identify the true hits and reduce the errors [43]. After finding its interactions with bacoside A and A_3_, we have found out that both plugs into a hydrophobic pocket of TPH. Further, our analysis showed that all the three docking algorithms identified similar residues (Tyr235, Thr265, Pro268, His272, Glu317, Phe318, Ser337, Glu340, Ile 366) surrounding (within 3.0 Å) bacoside A as best hit and interacts with different residues of TPH. However, HexDock and PatchDock (Fig. 1b) identified that Thr265 (1.46 and 1.91 Å) is the residue involved in hydrogen bond, where as AutoDock identified Glu 317 (1.79 Å) as an interacting residue (Fig. 1). Bacoside A – TPH complex showed docking score of 5002 with atomic contact energy −291.73. In addition, the estimated free energy for the complex is −9.29 kcal/mol with an inhibition constant (Ki) of 156.17 nM. Similarly, all the three docking algorithms showed Tyr235, Thr265, Pro268, Glu317, Ser337 and Cys364 residues (within 3.0 Å) in the pocket of the TPH surrounding bacoside A_3_. Among them all the three docking algorithm identified different residues Thr265 (1.91 Å) (Fig. 2a), Tyr235 (2.96 Å) (Fig. 2b) and Glu317 (2.48 Å) (Fig. 2c) interacting with bacoside A_3_ through hydrogen bond. Bacoside A_3_ with TPH complex showed docking score of 5472 with atomic contact energy −424.37. Further, we noted −10.23 kcal/mol as a estimated free energy for bacoside A_3_-TPH complex with the Ki of 37.36 nM.

In the present study, our in silico analysis showed that bacosides (A_3_, A) interact with TPH. We found that the bacoside A_3_ showed higher interaction score when compared to the interaction of bacoside A with TPH. The bioactive compounds: bacoside A and A_3_ formed hydrogen bond with TPH at two key residues Glu317 and Tyr235. Its note worthy to mention that tryptophan binds with Leu124-Asp139 and Ile367-Thr369 loop regions of TPH which are involved in conformational change [44, 45]. Interestingly, except Glu317 (O of Glu317 with H- of bacosides), the bacosides binding amino acids were not present at either the tryptophan or the co-factor BH4 (Gly 234, Leu 236, His 251, Glu 273, Glu 317) binding sites [44, 45]. In fact, we found that bacoside A_3_ and A formed hydrogen bond with Glu317. Although Glu317 of TPH bound with Fe, it also interacts with bacoside A_3_/A at a different side chain. Notably, bacoside A, A_3_ interact with Thr265, which is in the line of active site channel of TPH (residue 263–269 and 363–372) having high B-factor and less electron density [39]. It appears that these regions are highly flexible and the interaction at this site might enhance the TPH activity, possibly the reason for the observed elevated level of TPH activity [24]. Further, our analysis showed that bacoside A_3_ interacts with Tyr235 of TPH, which is conserved in all known TPH. Specific mutants of Tyr235 increase the *Km* for substrate (l-Trp) and lower *Km* for cofactor (BH4) [46]. These interactions add additional insight into the function of bacoside in 5-HT synthesis.

## Conclusion

Our in silico analysis showed that the bacosides (A_3_, A)-TPH complex interact through hydrogen bond with specific sites. These specific sites are known to regulate the catalytic mechanism of TPH [44–46], bacosides interactions may facilitate TPH activity and thus enhance the synthesis of serotonin [24]. Further study in bacosides interaction with different mutated TPH may provide greater insight in understanding the effect of bacosides in serotonergic system for the potential applications.

## General Experimental Procedures

The PDB format of TPH enzyme (PDB ID: 1MLW) was retrieved from protein data bank. Likewise, the chemical structures of bioactive compounds Bacoside A (Compound ID: 53398644), Bacoside A_3_ (Compound ID: 44152167) were collected from database (http://pubchem.ncbi.nlm.nih.gov) and then converted as protein data using OpenBabel [47] and docked with TPH. Different algorithms such as AutoDock [48], PatchDock [49] and HexDock [50] was used based on complementary shape principles matching two molecules by searching complementary to each other. Any type molecules (proteins, DNA, peptides, drugs) can be docked in PatchDock. A wide interface is ensured to include several matched local features of the docked molecules that have complementary characteristics. The molecules detected as concave are matched with convex and flat patches. Then the candidate transformation generated by patched with complementary patches. Each candidate transformation is further evaluated by a scoring function that considers both geometric fit and atomic desolvation energy. Finally, an RMSD (root mean square deviation) clustering is applied to the candidate solutions to discard redundant solutions [40]. The remaining matched candidates are ranked according to the geometric shape complementarity score. The generated TPH and bacoside three-dimension (3D) molecule complex was further analyzed by the Swiss-pdb viewer (ver 4.1). An algorithm has been used to identify the hydrophobic and hydrogen bond interaction, based on the distance between ligand–protein atoms, which is below 3.0 Å [51].
